# Perfluorinated Compounds in Umbilical Cord Blood and Adverse Birth Outcomes

**DOI:** 10.1371/journal.pone.0042474

**Published:** 2012-08-03

**Authors:** Mei-Huei Chen, Eun-Hee Ha, Ting-Wen Wen, Yi-Ning Su, Guang-Wen Lien, Chia-Yang Chen, Pau-Chung Chen, Wu-Shiun Hsieh

**Affiliations:** 1 Institute of Occupational Medicine and Industrial Hygiene, National Taiwan University College of Public Health, Taipei, Taiwan; 2 Department of Pediatrics, Cardinal Tien Hospital Yonghe Branch, New Taipei, Taiwan; 3 Department of Preventive Medicine, Ewha Womans University School of Medicine, Seoul, Korea; 4 Department of Medical Genetics, National Taiwan University Hospital, Taipei, Taiwan; 5 Institute of Clinical Genomics, National Taiwan University College of Medicine, Taipei, Taiwan; 6 Institute of Environmental Health, National Taiwan University College of Public Health, Taipei, Taiwan; 7 Department of Public Health, National Taiwan University College of Public Health, Taipei, Taiwan; 8 Department of Environmental and Occupational Medicine, National Taiwan University Hospital and National Taiwan University College of Medicine, Taipei, Taiwan; 9 Department of Pediatrics, National Taiwan University Hospital and National Taiwan University College of Medicine, Taipei, Taiwan; Stony Brook University, United States of America

## Abstract

**Background:**

Previous animal studies have shown that perfluorinated compounds (PFCs) have adverse impacts on birth outcomes, but the results have been inconclusive in humans. We investigated associations between prenatal exposure to perfluorooctanoic acid (PFOA), perfluorooctyl sulfonate (PFOS), perfluorononanoic acid (PFNA), and perfluoroundecanoic acid (PFUA) and birth outcomes.

**Methods:**

In total, 429 mother-infant pairs were recruited from the Taiwan Birth Panel Study (TBPS). Demographic data were obtained by interviewing mothers using a structured questionnaire and birth outcomes were extracted from medical records. Cord blood was collected for PFOA, PFOS, PFNA, and PFUA analysis by ultra-high-performance liquid chromatography/tandem mass spectrometry.

**Results:**

The geometric mean (standard deviation) levels of PFOA, PFOS, PFNA, and PFUA in cord blood plasma were 1.84 (2.23), 5.94 (1.95), 2.36(4.74), and 10.26 (3.07) ng/mL, respectively. Only PFOS levels were found to be inversely associated with gestational age, birth weight, and head circumference [per ln unit: adjusted *β* (95% confidence interval, CI) = −0.37 (−0.60, −0.13) wks, −110.2 (−176.0, −44.5) gm and −0.25 (−0.46, −0.05) cm]. Additionally, the odds ratio of preterm birth, low birth weight, and small for gestational age increased with PFOS exposure [per ln unit: adjusted odds ratio (OR) (95%CI) = 2.45 (1.47, 4.08), 2.61(0.85, 8.03) and 2.27 (1.25, 4.15)]. When PFOS levels were divided into quartiles, a dose-response relation was observed. However, PFOA, PFNA, and PFUA were not observed to have any convincing impact on birth outcomes.

**Conclusions:**

An adverse dose-dependent association was observed between prenatal PFOS exposure and birth outcomes. However, no associations were found for the other examined PFCs.

## Introduction

Perfluorinated compounds (PFCs) are persistent organic pollutants consisting of a carbon backbone, typically 4–14 carbons long, and a charged function moiety. Since they were first produced in the 1950 s, PFCs have been used in a variety of consumer and industrial products [1]. Health concerns to both wildlife and humans stem from the environmental accumulation of PFCs due to their chemical stabilities and resistance to biodegradation. During recent decades, efforts have been made globally to eliminate environmental pollutants, including eight-carbon PFCs, such as perfluorooctanoic acid (PFOA) and perfluorooctyl sulfonate (PFOS) [2], [3]. Nevertheless, PFCs are widely detectable in surface water, soil, ice caps, wild animals, and humans [1]. Furthermore, the consumption of alternative PFCs is unregulated. Recent research has revealed that increased perfluoralkyl chain length reduces the PFC elimination rate and increases PFC accumulation in the mouse liver [4]. PFCs elicited increased mouse and human peroxisome proliferator activated receptor-alpha (PPARα) activity with increasing carbon chain length up to 8–9 carbons; however, other PFCs with chain lengths longer than 9 carbons are less effective in their ability to activate PPARα [5]. The toxicological evidence on PFCs with other than 8 carbons is still limited.

PFCs have been reported to exhibit developmental toxicity in addition to hepatotoxicity, immunotoxicity, hormone disturbances, and tumorigenic potential [1]. In rodents, gestational exposure to PFOA, PFOS, or perfluorononanoic acid (PFNA) was found to be associated with fetal mortality, birth weight reduction, and delays in maturation [6]–[8]. However, the results of human epidemiologic studies are equivocal. For example, it has been reported that prenatal exposure to PFOA [9]–[11] and PFOS [9], [12] adversely affects birth outcomes, whereas other studies found no such association [13]–[20]. While the influence of prenatal PFCs exposure on commonly measured birth outcomes, such as gestational age, birth weight and birth length, was the focus of the previously mentioned studies, its impact on clinical diseases, such as preterm birth or small for gestational age, deserve more attention. Birth outcomes, such as small for gestational age and low birth weight, not only affect neonatal survival and childhood morbidity but also may be linked to adult diseases, such as diabetes, hypertension, and ischemic heart disease [21]. Therefore, investigations are required on the developmental toxicity of PFCs in humans, especially on the effects of PFCs on clinical diseases.

Maternal-fetal PFCs transfer had been documented in animal models and human studies [6], [7], [10]. The estimated mean half-lives of PFOA and PFOS in adults are 2.3 and 5.4 years, respectively [22], [23]. Accordingly, a single measurement of PFCs in cord blood should be sufficient to reflect the steady exposure status during the entire pregnancy. Therefore, in the present study, we used PFC levels in cord blood plasma to represent *in utero* exposure. Participants were recruited from a longitudinal birth cohort established between 2004 and 2005 in northern Taiwan, and their samples were analyzed for the following four PFCs: PFOA and PFOS (both 8-carbon products), PFNA (a 9-carbon product), and perfluoroundecanoic acid (PFUA; an 11-carbon product). The impacts of these four PFCs on birth outcomes, including gestational age, birth weight, birth length, head circumference, Ponderal index, preterm birth, and small for gestational age, were investigated.

## Materials and Methods

### Ethic Statement

Mothers provided written informed consent for themselves and their babies to participate in the study. The Research Ethics Committee of Taiwan National University Hospital approved the protocol. All aspects of data collection and storage were in accordance with the standards stipulated by this approval.

### Study Population

The study subjects were from the Taiwan Birth Panel Study (TBPS), a longitudinal birth cohort study that was conducted at one medical center in Taipei and one local hospital and two clinics in New Taipei from April 2004 to January 2005. Mothers were interviewed by trained interviewers using a structured prenatal questionnaire during the postpartum hospital stay. Cord blood was collected at birth and stored at −80°C until required for laboratory analysis. A total of 439 mother-infant pairs were initially enrolled. Infants with incomplete records of birth weight (n = 2) and birth length (n = 2) were excluded. Furthermore, 5 active smokers and 1 mother with unknown environmental tobacco smoke (ETS) exposure status during pregnancy were also excluded. Finally, 429 mother-infant pairs constituted the study cohort.

### Exposure Assessment

Our previous study had developed a fast and sensitive ultra-high-performance liquid chromatography/tandem mass spectrometry method using the Waters ACQUITY UPLC system (Waters Corporation, Milford, MA, USA) coupled with a Waters Quattro Premier XE triple quadrupole mass spectrometer for the determination of twelve PFCs in cord blood [24]. Of these, four PFCs (PFOA, PFOS, PFNA and PFUA) with detection rates above 60% were selected for analysis in this study, and their limits of quantitation (LOQs) were 1.58, 0.22, 0.84, and 3.1 ng/mL, respectively. All of the laboratory analyses were conducted by investigators blinded to the characteristics of study subjects. Among 429 cord blood samples, the detection rates of PFOA, PFOS, PFNA, and PFUA were 83.7%, 100%, 68.5% and 85.5%, respectively. Consequently, values < LOQs were assumed to be half the appropriate LOQ level.

### Outcome Variables

The birth outcomes examined in this study were the following: gestational age (weeks), birth weight (grams), birth length (centimeters), head circumference (centimeters), Ponderal index (gm/cm^3^), preterm birth, low birth weight and small for gestational age at birth. All of the data were extracted from medical records. Gestational age was assessed by obstetricians based on last menstruation or early ultrasound estimates; other measurements were obtained by trained nurses. The Ponderal index was calculated by dividing birth weight (grams) by the third power of birth length (centimeters) and multiplying the result by 100. Preterm birth was defined as a gestational age <37 weeks. Low birth weight was defined as a birth weight <2,500 gm. A birth weight below the 10^th^ percentile for gestational age was classified as small for gestational age. Birth weight for gestational age was determined using the percentile scale derived from national Taiwanese data [25].

### Potential Confounders

The cotinine level in umbilical cord blood, an exposure index of prenatal ETS exposure, was analyzed by high-performance liquid chromatography (PerkinElmer, Boston, MA, USA) coupled to a triple quadruple tandem spectrometer (API 3000TM, Applied Biosystems, USA). The limit of detection of cord blood cotinine was 0.05 ng/mL. The original levels were natural log transformed due to a right-skewed distribution.

The other potential confounders were obtained using the structured prenatal questionnaire. These confounders were selected a priori according to the reported literature [9]–[16], including maternal age at conception, prepregnancy body mass index (BMI), educational level, log (Ln)-transformed cord blood cotinine levels, type of delivery, infant sex and parity. Because advanced maternal age was associated with a higher risk of adverse pregnancy outcomes, mothers were classified into two groups by their age: <35 and ≥35 years. The BMI was calculated as the body weight divided by the height squared according to WHO standards. Prepregnancy BMIs were classified into 3 groups: underweight (BMI<18.5 kg/m^2^), normal weight (BMI 18.5–24.9 kg/m^2^), and overweight (BMI ≥25.0 kg/m^2^).

### Statistical Analysis

Initially, the distributions of PFC levels in mothers and infants with different characteristics were compared using Wilcoxon’s rank sum test and the Kruskal Wallis test. The levels of PFOA, PFOS, PFNA, and PFUA were then natural log transformed due to a right-skewed distribution. Correlations between the levels of the four PFCs were determined using Pearson’s correlation, and correlation coefficients were <0.45; thus, their influences on birth outcomes were analyzed separately. Simple and multiple linear regression models were used to explore the associations between PFCs levels in cord blood plasma and gestational age, birth weight, length, head circumference, and Ponderal index. Additionally, the influence of PFCs levels on preterm birth, low birth weight and small for gestational age was investigated by logistic regression. The potential confounders, namely maternal age, prepregnancy BMI, education level, log (Ln)-transformed cord blood cotinine levels, type of delivery, parity and infant sex, were included in the adjusted model. Because gestational age is considered to be the major influential factor for birth outcomes, gestational age was added to the adjusted model for birth outcomes for birth weight, birth length, head circumference, Ponderal index and low birth weight. Finally, we categorized PFOS levels into quartiles (0.11 to 3.84, 3.85 to 5.67, 5.68 to 8.82 and 8.82 to 67.9 ng/mL) and set the lowest level as a reference. The dose-response relationship between PFOS levels and birth outcomes was assessed using the same adjusted model. In addition, regression diagnosis was performed to identify influential points using Cook’s distance. Because the estimate was similar when these points were excluded from the analysis, only the results of all participants are presented. Statistical analyses were performed using SAS version 9.1 (SAS Institute Inc., Cary, NC, USA). All of the tests were two-sided, and statistical significance was accepted for *P* values of <0.05.

## Results

There were 429 mother-infant pairs in total, and the geometric mean (standard deviation) levels were 1.84(2.23), 5.94(1.95), 2.36(4.74), and 10.26 (3.07) ng/mL for PFOA, PFOS, PFNA, and PFUA, respectively (Table 1). Regarding maternal age and prepregnancy BMI, the levels of PFOA, PFOS, PFNA, and PFUA were similar between subgroups. PFOS levels in umbilical cord blood plasma were higher for mothers with less than 12 years of education and mothers who delivered via cesarean section. Higher PFOA and PFNA levels were found in those with higher cord blood cotinine levels. For infants, the levels of PFCs did not differ by sex, except PFUA, which showed a higher level in girls. Levels of PFOA and PFUA decreased significantly as parity increased. The prevalence rates of preterm birth, low birth weight, and small for gestational age were 9.3%, 6.1%, and 6.1%, respectively.

**Table 1:** Concentrations of perfluorinated compounds [geometric mean (geometric standard deviation) ng/mL] in cord blood plasma by maternal and infant characteristics.

Characteristics^a^	%	PFOA^b^	PFOS	PFNA^b^	PFUA^b^
**Total**	100.0	1.84 (2.23)	5.94 (1.95)	2.36 (4.74)	10.26 (3.07)
**Mother**					
Age (years)					
<35	77.2	1.77 (2.16)	5.84 (1.95)	2.49 (4.69)	10.58 (3.14)
≥35	22.8	2.11 (2.46)	6.31 (1.94)	1.97 (4.90)	9.25 (2.86)
Prepregnancy BMI (kg/m^2^)					
<18.5	14.7	1.96 (2.33)	5.64 (1.93)	2.43 (5.12)	10.19 (3.51)
18.5–24.9	78.8	1.80 (2.21)	6.02 (1.95)	2.35 (4.73)	10.11 (3.01)
>25.0	6.5	2.19 (2.39)	5.75 (1.93)	2.34 (4.35)	12.41 (2.93)
Education (years)					
≤12	55.0	1.76 (2.16)	6.44 (1.95)**	2.49 (4.62)	10.54 (3.30)
>12	45.0	1.95 (2.32)	5.38 (1.92)**	2.21 (4.90)	9.92 (2.80)
Cord blood cotinine (ng/mL)					
<0.25	49.7	1.60 (2.14)***	5.68 (2.03)	1.87(4.52)**	10.52(3.01)
≥0.25	50.3	2.12 (2.28)***	6.21(1.95)	2.96(4.83)**	10.00(3.15)
Type of delivery					
Vaginal	59.4	1.87 (2.22)	5.46 (1.89)**	2.53 (4.82)	10.01 (3.14)
Cesarean section	40.6	1.80 (2.26)	6.73 (1.99)**	2.12 (4.63)	10.63 (2.98)
**Infant**					
Sex					
Boy	52.2	1.84 (2.20)	6.00 (2.07)	2.25 (4.84)	8.72 (3.20)**
Girl	47.8	1.85 (2.27)	5.88 (1.81)	2.48 (4.65)	12.25 (2.86)**
Parity					
0	48.3	2.00 (2.19)*	5.68 (1.95)	2.73 (4.76)	11.92 (2.97)*
≥1	51.7	1.71 (2.26)*	6.20 (1.93)	2.06 (4.69)	8.91 (3.12)*
Preterm					
No	90.7	1.88 (2.26)	5.71 (1.92)***	2.40 (4.77)	10.40 (3.05)
Yes	9.3	1.49 (1.97)	8.85 (1.93)***	1.99 (4.51)	8.99 (3.33)
Low birth weight					
No	93.9	1.87 (2.24)	5.75 (1.92)**	2.42 (4.73)	10.29 (3.08)
Yes	6.1	1.51 (2.04)	9.92 (2.01)**	1.56 (4.84)	9.70 (3.08)
Small for gestational age					
No	93.9	1.83 (2.22)	5.81 (1.89)	2.38 (4.66)	10.30 (3.07)
Yes	6.1	2.04 (2.56)	8.47 (2.60)	2.06 (6.33)	9.60 (3.20)

Abbreviations: BMI, body mass index; PFNA, perfluorononanoic acid; PFOA, perfluorooctanoic acid; PFOS, perfluorooctyl sulfonate; PFUA, perfluoroundecanoic acid.

* *P*<0.05; **P<0.01; ***P<0.001 (*P* value are 2 sided).

a There were 429 subjects in total.

b For PFOA, PFNA and PFUA, concentration value below LOQ were set to be 1/2 LOQ.

Table 2 shows the distribution of birth outcomes for this study population. The mean gestational age was 38.5 weeks and ranged from 29.0 to 41.0 weeks. The means and standard deviations were 3,166.7 (473.0) gm for birth weight, 49.0 (2.20) cm for birth length, 33.6 (1.63) cm for head circumference, and 2.67 (0.24) gm/cm^3^ for Ponderal index.

**Table 2:** Distributions of birth outcomes.

Birth outcomes	Mean (SD)	Median	Range
Gestational age (weeks)	38.5 (1.69)	39.0	29.0–41.0
Birth weight (gm)	3166.7 (473.02)	3164	1024–5100
Birth length (cm)	49.0 (2.20)	49.0	37.0–57.0
Head circumference (cm)	33.6 (1.63)	34.0	25.0–37.0
Ponderal index^a^ (gm/cm^3^)	2.67 (0.24)	2.67	1.84–3.48

a Ponderal index: birth weight (gram) divide by third power of body length (centimeter), then multiplied by 100.

The associations between PFCs levels in cord blood plasma and birth outcomes are displayed in Table 3. In the crude linear regression model, PFOS levels were inversely associated with gestational age, birth weight, birth length, and head circumference. After adjusting for potential confounders, the adverse effect of PFOS on gestational age, birth weight, and head circumference remained statistically significant [per ln unit: *β* (95% confidence interval, CI) = −0.37 (−0.60, −0.13) for gestational age, −110.2 (−176.0, −44.5) for birth weight and −0.25 (−0.46, −0.05) for head circumference]. The β coefficients of LnPFOA for birth outcomes in both crude and adjusted models were mostly negative, except for gestational age, and did not reach statistical significance. A non-significant inverse association was also observed between LnPFUA and birth outcomes, except for birth length. For LnPFNA, β coefficients were mostly positive, except for Ponderal index, in both the crude and adjusted models. Significance was observed for birth length [per ln unit: *β* (95% CI) = 0.16 (0.05, 0.27)] and Ponderal index [per ln unit: *β* (95% CI) = −0.02 (−0.03, −0.004)].

**Table 3:** Regression coefficients and 95% confidence intervals of multiple linear regression model of birth outcomes by perfluorinated compounds in cord blood.

Birth outcomes	LnPFOA^a^ (ng/mL)	LnPFOS (ng/mL)	LnPFNA^a^ (ng/mL)	LnPFUA^a^ (ng/mL)
	β coefficient (95% confidence interval)			
Gestational age (wks)				
crude	0.06	−0.49***	0.03	−0.04
	(−0.14, 0.26)	(−0.73, −0.25)	(−0.07, 0.14)	(−0.19, 0.10)
adjusted^b^	0.06	−0.37**	0.04	−0.05
	(−0.14, 0.26)	(−0.60, −0.13)	(−0.06, 0.14)	(−0.20, 0.09)
Birth weight (gm)				
crude	−14.2	−118.8***	6.09	−42.3*
	(−69.8, 41.4)	(−185.4, −52.1)	(−22.9, 35.1)	(−82.2, −2.49)
adjusted^b^	−19.2	−110.2**	6.07	−24.7
	(−63.5, 25.1)	(−176.0, −44.5)	(−16.6, 28.7)	(−56.0, 6.59)
Birth length (cm)				
crude	0.01	−0.51**	0.16*	−0.06
	(−0.25, 0.27)	(−0.82, −0.19)	(0.03, 0.30)	(−0.25, 0.13)
adjusted^b^	−0.003	−0.17	0.16**	0.005
	(−0.21, 0.21)	(−0.42, 0.09)	(0.05, 0.27)	(−0.14, 0.16)
Head circumference (cm)				
crude	−0.006	−0.42***	0.03	−0.12
	(−0.20, 0.19)	(−0.65, −0.19)	(−0.07, 0.13)	(−0.26, 0.02)
adjusted^b^	−0.05	−0.25*	0.05	−0.05
	(−0.22, 0.17)	(−0.46, −0.05)	(−0.04, 0.13)	(−0.17, 0.07)
Ponderal index^c^ (gm/cm^3^)				
crude	−0.01	−0.03	−0.02*	−0.02*
	(−0.04, 0.02)	(−0.06, 0.006)	(−0.03, −0.004)	(−0.04, −0.0003)
adjusted^b^	−0.01	−0.01	−0.02*	−0.01
	(−0.04, 0.02)	(−0.05, 0.02)	(−0.03, −0.004)	(−0.03, 0.005)

Abbreviations: PFNA, perfluorononanoic acid; PFOA, perfluorooctanoic acid; PFOS, perfluorooctyl sulfonate; PFUA, perfluoroundecanoic acid.

* *P*<0.05; **P<0.01; ***P<0.001 (*P* value are 2 sided).

a For PFOA, PFNA and PFUA, concentration value below LOQ were set to be 1/2 LOQ.

b Model adjusted for maternal age, prepregnancy body mass index, education level, log (Ln)-transformed cord blood cotinine levels, type of delivery, parity and infant sex and gestational age for birth weight, birth length, head circumference, and Ponderal index.

c Ponderal index: birth weight in grams divide by third power of body length in centimeters, then multiplied by 100.

Table 4 shows the effect of prenatal PFCs exposure on preterm birth, low birth weight, and small for gestational age. Similar to the linear regression model results in Table 3, PFOS had obvious and consistent influences on birth outcomes. The odds ratio (OR) of preterm birth and small for gestational age increased significantly in both crude and adjusted model [per ln unit: OR (95% CI) = 2.45 (1.47, 4.08) for preterm birth and 2.27 (1.25, 4.15) for small for gestational age]. However, the OR of low birth weight was only significant in the crude model [per ln unit: OR (95% CI) = 3.10 (1.77, 5.43) in crude model and 2.61 (0.85, 8.03) in adjusted model]. For PFOA, PFNA and PFUA, no significant associations were found between levels in cord blood plasma and birth outcomes.

**Table 4:** Odds ratio and 95% confidence intervals of logistic regression model of birth outcomes by perfluorinated compounds in cord blood.

Birth outcome	LnPFOA^a^ (ng/mL)	LnPFOS (ng/mL)	LnPFNA^a^ (ng/mL)	LnPFUA^a^ (ng/mL)
	odds ratio (95% confidence interval)			
Preterm				
crude	0.69	2.59***	0.93	0.89
	(0.45, 1.06)	(1.61, 4.16)	(0.75, 1.14)	(0.67, 1.19)
adjusted^b^	0.64	2.45***	0.88	0.87
	(0.40, 1.02)	(1.47, 4.08)	(0.71, 1.11)	(0.64, 1.16)
Low birth weight				
crude	0.71	3.10***	0.83	0.95
	(0.42, 1.20)	(1.77, 5.43)	(0.64, 1.08)	(0.67, 1.35)
adjusted^b^	0.53	2.61	0.76	1.01
	(0.18, 1.55)	(0.85, 8.03)	(0.47, 1.23)	(0.53, 1.91)
Small for gestational age				
crude	1.18	2.22**	0.94	0.95
	(0.73, 1.91)	(1.27, 3.88)	(0.73, 1.22)	(0.67, 1.34)
adjusted^b^	1.24	2.27**	0.97	0.93
	(0.75, 2.05)	(1.25, 4.15)	(0.74, 1.26)	(0.65, 1.33)

Abbreviations: PFNA, perfluorononanoic acid; PFOA, perfluorooctanoic acid; PFOS, perfluorooctyl sulfonate; PFUA, perfluoroundecanoic acid.

* *P*<0.05; **P<0.01; ***P<0.001 (*P* value are 2 sided).

a For PFOA, PFNA and PFUA, concentration value below LOQ were set to be 1/2 LOQ.

b Model adjusted for maternal age, prepregnancy body mass index, education level, log (Ln)-transformed cord blood cotinine levels, type of delivery, parity and infant sex and gestational age for low birth weight.

**Figure 1:**
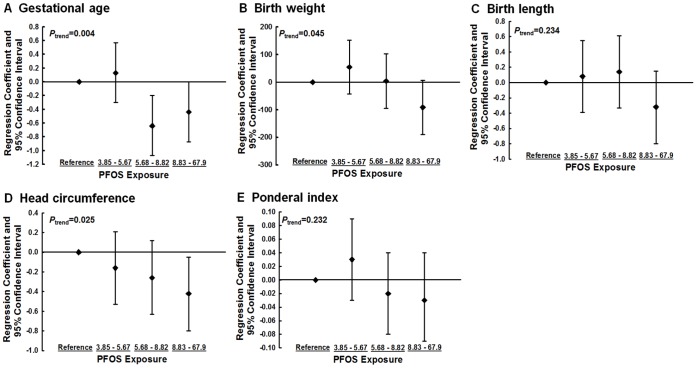
Adjusted regression coefficients and 95% confidence intervals of birth outcomes by PFOS levels (in quartiles, ng/mL) in cord blood plasma. Birth outcomes are shown as (A) gestational age (wks), (B) birth weight (gm), (C) birth length (cm), (D) head circumference (cm) and (E) Ponderal index (gm/cm^3^). Models were adjusted for maternal age, prepregnancy body mass index, education level, log (Ln)-transformed cord blood cotinine levels, type of delivery, parity and infant sex and gestational age for birth weight, birth length, head circumference, and Ponderal index. The lowest quartile (PFOS levels between 0.11 and 3.84 ng/mL) was set as a reference.

**Figure 2:**
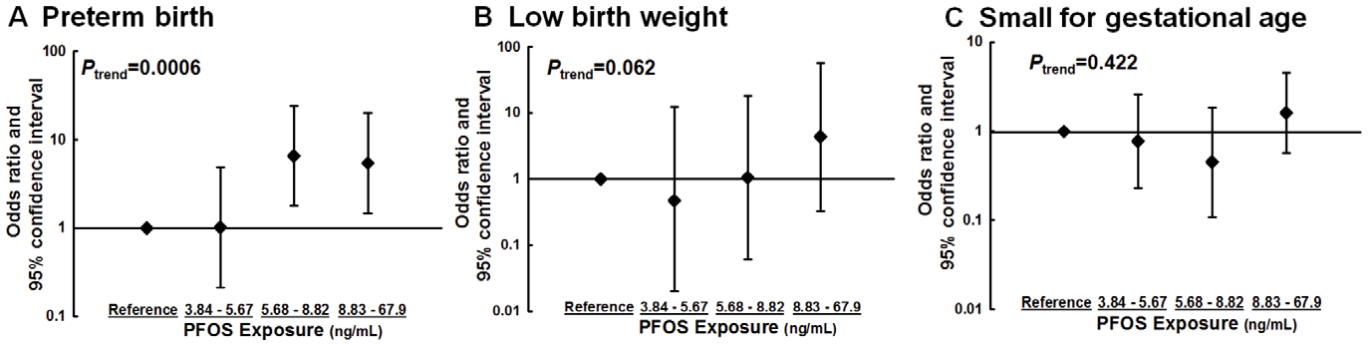
Adjusted odds ratio and 95% confidence intervals of birth outcomes by PFOS levels (in quartiles, ng/mL) in cord blood plasma. Birth outcomes are shown as (A) preterm birth, (B) low birth weight, and (C) small for gestational age. Models were adjusted for maternal age, prepregnancy body mass index, education level, log (Ln)-transformed cord blood cotinine levels, type of delivery, parity and infant sex and gestational age for low birth weight. The lowest quartile (PFOS levels between 0.11 and 3.84 ng/mL) was set as a reference.

The dose-response relationship between PFOS exposure and birth outcomes is presented in the figures. The PFOS levels were divided into quartile, and Figure 1 shows the adjusted regression coefficients and 95% CI of birth outcomes, and Figure 2 shows the adjusted OR and 95% CI of birth outcomes. The significance of *P* for trend was noted in gestational age, birth weight, head circumference and preterm birth. Among other birth outcomes, such as birth length, Ponderal index, small for gestational age and low birth weight, the effect of PFOS levels was the greatest in the highest exposure group compared with other exposure groups.

## Discussion

This study showed that PFOS levels in cord blood plasma are negatively associated with gestational age, birth weight, head circumference, preterm birth, and small for gestational age. After classifying the PFOS levels into quartiles, a dose-response relation was still observed among most of these birth outcomes. Among PFCs with chain lengths longer than 8 carbons, PFNA and PFUA were commonly detectable in this study population. However, we did not find a convincing association between birth outcomes and prenatal exposure to PFOA, PFNA or PFUA.

Animal studies in rodents have shown that *in utero* PFOS exposure can lead to increased fetal mortality and decreased body size [1], [6], [7]. Our study results are consistent with these findings, but our study subjects were exposed to levels far below the levels reported in animal studies. For prenatal PFOS exposure in rats, the lower 95% confidence limit of the benchmark dose of a 5% response (BMDL_5_) for decreased gestational length and birth weight effect was estimated to be 0.31 mg/kg/day and 0.39 mg/kg/day, respectively [7]. Species differences in elimination time, metabolic pathways, and susceptibility could contribute to this discrepancy [1], [22]. While investigating the dose-response relationship between prenatal PFOS exposure and birth outcomes, this study found that the highest exposure group had the greatest adverse effects on birth outcomes. The statistical insignificance in analysis could be due to the imitations of population size or the phenomenon of critical body burden [26]. However, the mechanism through which PFOS impairs fetal growth remains unclear. Studies on peroxisome proliferator activated receptor-alpha (PPARα) knockout mice revealed increased neonatal lethality and weight deficits for PFOA and PFNA but not PFOS [8], [27], [28]. As a weaker PPARα agonist, PFOS might interfere with fetal development via another pathway.

The relationship between *in utero* PFCs exposure and birth outcomes in humans is unclear [9]–[20], [29]. Occupational studies without biological measurements were not able to identify a significant correlation between PFOS exposure and birth weight or pregnancy outcome [17], [18]. However, most of the epidemiological studies with direct measurements of exposure did demonstrate increased adverse effects of prenatal PFCs exposure on birth outcomes [9]–[16]. A comparison of previous studies related to PFOS or PFOA exposure and birth outcomes among the general populations is shown in Table S1 and Table S2 [9]–[16]. All of the studies investigated the impact of prenatal PFOS and PFOA exposure on birth weight and revealed either negative associations or null effects. The results were similar for birth outcomes, such as birth length, head circumference and Ponderal index. However, their influence on gestational age, preterm birth, low birth weight and small for gestational age was more diverse. Discrepancies might be related to methodological differences, the characteristics of study subjects, the prevalence of preterm birth or small for gestational age and unmeasured confounders [29]. Moreover, different degrees of the impact of PFCs on gestational length and body size may influence the current discovery.

Our study results are similar to those of a Japanese study [12]. Prenatal PFOS but not PFOA exposure had significant adverse impacts on birth outcomes. In studies conducted in the United States and Denmark, significant results were observed for prenatal PFOA exposure [9]–[11]. The susceptibility may differ from race to race. In this study, the adverse impact of prenatal PFOS exposure was consistent among all birth outcomes. Gestational age and other potential confounders were taken into consideration. Accordingly, we believe that our results identify toxicological differences between PFCs. A 100 gm reduction in birth weight could be a minor change for a newborn but could lead to left shifting of the normal birth weight distribution in the overall population. This phenomenon is related to the increased proportion of low birth weight or small for gestational age babies who exhibit higher morbidity and mortality. Because PFOS is commonly detectable in the general population, its influence on birth outcomes should not be neglected.

Few studies have addressed the developmental toxicities of PFNA and PFUA. These two entities are PFCs with 9 and 11 carbon chains, respectively. According to the reported literature, PFCs with longer carbon chains exhibit slower urinary elimination and increased peroxisomal β-oxidase activity and accumulation in the mouse liver [4]. For PFNA, developmental toxicity was found to occur via PPARα in mice [8]. Moreover, even though a study of 101 pregnant women in Canada failed to reveal any significant impact of *in utero* PFNA exposure on birth weight [14], PFNA was found to be positively associated with birth length and negatively associated with Ponderal index in the present study. However, in the present study, the impact of PFNA on all birth outcome variables was not consistent, and the detection rate and exposure levels of PFNA were different from those found in previous reports [14]. Accordingly, our results require careful interpretation. No previous study has reported an association between prenatal PFUA exposure and birth outcomes. The geometric mean concentration of PFUA in cord blood was higher (10.26 ng/mL) in the present study than in a Baltimore study (upper range of 1.90 ng/mL) [30]. However, we found no significant association between PFUA and birth outcomes after adjusting for potential confounders. Furthermore, the developmental toxicities of PFCs with longer perfluoralkyl chains need to be clarified.

The levels of PFOA and PFOS in the present study differed from literature values (Table S1 and Table S2). The study years (from1996 to 2006) could lead to some differences in exposure levels because the global action toward the phasing out of these two chemicals began in the early 2000 s [2], [3]. The concentration of PFCs in maternal blood and cord blood was highly correlated [10]. Roughly, the proportion of PFOS in cord blood was one-third of maternal blood and was two-thirds for PFOA. The geometric mean levels of PFOA and PFOS in the present study are lower than those of Fei et al. and higher than Washino et al., although they are similar to other studies [9]–[16]. Differences in study location, blood collection times, and analytic techniques could contribute to this non-agreement. Nevertheless, some potential exposure sources need to be mentioned. First, the concentrations of PFOS and PFOA in rivers near semiconductor and electronics industries in Taiwan are higher than other countries [31]. Second, studies have demonstrated detectable PFCs in nonstick coatings on cookware and in food packaging [32], [33]. In Taiwan, high-temperature frying and the packing of hot foods are common. Furthermore, diet and drinking water could contribute to PFCs exposure. In Taiwan, 14 types of food samples were analyzed, and PFOA was found to be the highest among the analytes [34]. The proportion of PFOS or PFUA intake from food was quite low. Thus, the exposure source of PFOS and PFUA requires further investigation.

In the present study, several maternal and infant characteristics were found to be associated with levels of PFCs. Consistent with previous reports, cord blood PFOS and PFOA levels were relatively unaffected by maternal age or prepregnancy BMI [9], [12]. However, levels of PFOA and PFUA in cord blood decreased with increasing parity, possibly because of changes in placental transfer characteristics [9], [10], [12]. In our study, a lower educational level and delivery by cesarean section were found to be associated with higher PFOS levels. The former could be related to a lower socioeconomic status, as suggested by Fei et al. [10], but the latter contradicts the findings of Washino et al [12]. Because of the high cesarean section rate in Taiwan, it is difficult to compare the results of high PFOS levels with previous reports.

Maternal smoking and exposure to ETS are well-known risk factors for adverse birth outcomes [35]. In this study, we excluded a small number of active smokers and measured cotinine levels in cord blood as a sensitive marker for ETS exposure. Cord blood cotinine and PFOS levels were not correlated. Cord blood cotinine levels had a significant inverse association with gestational age, birth weight and preterm birth (data not shown). Our study results were consistent with previous studies [35]. Therefore, log (Ln)-transformed cord blood cotinine levels were included as a potential confounder in the adjusted model. The influences of prenatal PFOS exposure were maintained.

The strength of this study is its prospective cohort design, which minimizes recall bias, and the extraction of growth measurements from reliable medical records. Furthermore, the impacts of four PFCs, including products with a longer perfluoroalkyl chain, were investigated. To our knowledge, this is the first report on the association between prenatal PFUA exposure and birth outcomes. However, this study has several potential limitations. First, the sample size was not large enough to form conclusions on the impacts of PFCs on birth outcomes with low prevalence rates, such as low birth weight or small for gestational age. Second, a lack of information concerning maternal diet habits could further limit the exploration of exposure sources, but would not alter the impact of prenatal PFOS exposure on birth outcomes.

In summary, higher PFOS levels in cord blood plasma were associated with adverse birth outcomes in a birth cohort in Taiwan. Longitudinal follow-up of the present study population could help clarify the long-term impact of PFCs on growth and the health effects of background exposure levels. Although the association between prenatal PFNA or PFUA and birth outcomes was not proven to be significant, the high detection rate and concentration of these two chemicals in cord blood plasma suggest human exposure, and the possibility of other health effects was not examined in this study.

## Supporting Information

Table S1 **Summary of previous studies of prenatal perfluorooctyl sulfonate (PFOS) exposure and birth outcomes among the general populations.**
(DOCX)

Table S2 **Summary of previous studies of prenatal perfluorooctanoic acid (PFOA) exposure and birth outcomes among the general populations.**
(DOCX)
