# Supplementary material for: Perfluorinated Compounds in Umbilical Cord Blood and Adverse Birth Outcomes
Source: PLoS One. 2012 Aug 3;7(8):e42474. doi: 10.1371/journal.pone.0042474 (PMC3411780; doi:10.1371/journal.pone.0042474)
Supplement: Table S2 — Summary of previous studies of prenatal perfluorooctanoic acid (PFOA) exposure and birth outcomes among the general populations. (DOCX) [file pone.0042474.s002.docx]

Table S2. Summary of previous studies of prenatal perfluorooctanoic acid (PFOA) exposure and birth outcomes among the general populations

| Author | Study years | Location | Sample size | Conc., ng/mL | Gestational age | Birth weight | Birth length | Head circumference | Ponderal index | Preterm birth | LBW | SGA |
| --- | --- | --- | --- | --- | --- | --- | --- | --- | --- | --- | --- | --- |
| Apelberg et al. [9] | 2004-2005 | United States | 293 | 1.6^b^ | + | − | ∅ | −^*^ | −^*^ |  |  |  |
| Fei et al. [10] | 1996- | Denmark | 1387 | 5.6^c^ | ∅ | −^*^ |  |  |  | ↑ | ↑ | ∅ |
| Fei et al. [11] | 2002 | Denmark | 1387 | 5.6^c^ |  |  | −^*^ | − | + |  |  |  |
| Monroy et al. [14] | 2004-2005 | Canada | 105 | 1.9^a^ |  | ∅ |  |  |  |  |  |  |
| Washino et al.  [12] | 2002-2005 | Japan | 428 | 1.4^c^ |  | − | − | − |  |  |  |  |
| Hamm et al. [15] | 2005-2006 | Canada | 252 | 2.1^c^ | ∅ | ∅ |  |  |  | ∅ |  | ↓ |
| Whitworth et al. [16] | 2003-2004 | Norway | 901 | 2.2^d^ |  | − |  |  |  | ↓^*^ |  | ∅ |
| Present study | 2004-2005 | Taiwan | 429 | 1.8^a^ | + | − | ∅ | − | − | ↓ | ↓ | ↑ |

Abbreviations: Conc., concentration; LBW, low birth weight; SGA, small for gestational age; ∅, represents an null effect; +, represents a positive association; −, represents a negative association; ↑, represents increased odds ratio; ↓, represents decreased odds ratio.

^*^*P*<0.05

^a^mean levels in cord blood ^b^median levels in cord blood

^c^mean levels in maternal blood ^d^median levels in maternal blood
